# MPK3/MPK6 are involved in iron deficiency-induced ethylene production in Arabidopsis

**DOI:** 10.3389/fpls.2015.00953

**Published:** 2015-11-03

**Authors:** Lingxiao Ye, Lin Li, Lu Wang, Shoudong Wang, Sen Li, Juan Du, Shuqun Zhang, Huixia Shou

**Affiliations:** ^1^State Key Laboratory of Plant Physiology and Biochemistry, College of Life Sciences, Zhejiang UniversityHangzhou, China; ^2^Key Laboratory of Tea Biology and Resources Utilization, Ministry of Agriculture, National Center for Tea Plant Improvement, Tea Research Institute, Chinese Academy of Agricultural SciencesHangzhou, China

**Keywords:** *Arabidopsis*, ethylene, Fe deficiency, mitogen-activated protein kinase (MPK), 1-Aminocyclopropane-1-carboxylic acid synthase (ACS)

## Abstract

Iron (Fe) is an essential micronutrient that participates in various biological processes important for plant growth. Ethylene production induced by Fe deficiency plays important roles in plant tolerance to stress induced by Fe deficiency. However, the activation and regulatory mechanisms of 1-Aminocyclopropane-1-carboxylic acid synthase (ACS) genes in this response are not clear. In this study, we demonstrated that Fe deficiency increased the abundance of *ACS2, ACS6, ACS7*, and *ACS11* transcripts in both leaves and roots as well as the abundance of *ACS8* transcripts in leaves and *ACS9* transcripts in roots. Furthermore, we investigated the role of mitogen-activated protein kinase 3 and 6 (MPK3/MPK6)-regulated ACS2/6 activation in Fe deficiency-induced ethylene production. Our results showed that MPK3/MPK6 transcript abundance and MPK3/MPK6 phosphorylation are elevated under conditions of Fe deficiency. Furthermore, *mpk3* and *mpk6* mutants show a lesser induction of ethylene production under Fe deficiency and a greater sensitivity to Fe deficiency. Finally, in *mpk3, mpk6*, and *acs2* mutants under conditions of Fe deficiency, induction of transcript expression of the Fe-deficiency response genes *FRO2, IRT1*, and *FIT* is partially compromised. Taken together, our results suggest that the MPK3/MPK6 and ACS2 are part of the Fe starvation-induced ethylene production signaling pathway.

## Introduction

Iron (Fe) is an essential micronutrient that plays an important role in plant growth. It participates in several metabolic processes including respiration, photosynthesis, and chlorophyll biosynthesis (Kobayashi and Nishizawa, 2012). Since Fe is poorly soluble in neutral or basic soils, it is not readily available to plants in these conditions (Kim and Guerinot, 2007). To counteract Fe deficiency, plants have developed a set of responses to control the uptake, utilization, and storage of Fe. Most plants, with the exception of those in the Graminaceae family, use strategy I, which is also known as the reduction strategy. Strategy I plants, via H^+^-ATPases, excrete protons into the rhizosphere to increase the solubility of Fe. Ferric-chelate reductase oxidases present on the root surface then reduce Fe^3+^ into Fe^2+^, after which Fe^2+^ transporters take Fe^2+^ into the plant. Consistent with the roles of these proteins in strategy I plants, in Arabidopsis, expression of the plasma membrane H^+^ ATPase (*AHA2*), the major ferric-chelate reductase oxidase (*FRO2*), and the major Fe^2+^ transporter (*IRT1*) is strongly induced under Fe deficiency (Eide et al., 1996; Robinson et al., 1999; Santi and Schmidt, 2009). The Graminaceae family plants use strategy II, which is also known as the chelate strategy (Kim and Guerinot, 2007; Walker and Connolly, 2008). Strategy II plants release phytosiderophores, which can directly bind Fe^3+^ (Conte and Walker, 2011), into the rhizosphere. The chelated complexes are then transported into the roots through the YS/YSL family of transporters (Curie et al., 2001, 2009).

Recent studies have shown that in strategy I plants, phytohormones such as ethylene, auxin, cytokinins, and nitric oxide (NO) are involved in the regulation of Fe deficiency responses (Romera et al., 2011; Ivanov et al., 2012). In particular, Fe deficiency increases production of ethylene in roots of strategy I plants (Romera et al., 1999; Romera and Alcantara, 2004). It is thought that ethylene regulates *FRO2* and *IRT1* gene expression through the modulation of the major transcription factor FER or FER-like (Lucena et al., 2006). In support of this, in conditions of Fe deficiency, expression of Arabidopsis *FIT*, which is homolog of tomato *FER* decreases upon inhibition of ethylene synthesis or activity, and increases upon addition of ethylene precursor (García et al., 2010). And the expression of Fe-related genes and ferric reductase activity were also induced by ethylene level (Romera and Alcantara, 1994; Li and Li, 2004; Lucena et al., 2006; Waters et al., 2007; García et al., 2010). The genes involved in ethylene biosynthesis and signaling could also be up-regulated under Fe deficiency (García et al., 2010). Ethylene biosynthesis involves three enzymatic steps: (1) S-AdoMet synthetase converts methionine to S-adenosyl-L-methionine (S-AdoMet); (2) S-AdoMet is converted to ACC by ACC synthase (ACS); (3) ACC is oxidized by ACC oxidase (ACO) and is thereby converted to ethylene (Yang and Hoffman, 1984; Sato and Theologis, 1989; Zarembinski and Theologis, 1994; Wang et al., 2002; Chae and Kieber, 2005). Unlike ACO, ACS has very low basal activity and can be rapidly increased under conditions that promote ethylene production (Yang and Hoffman, 1984). Thus, ACS is considered to be the rate-limiting enzyme in ethylene biosynthesis.

Arabidopsis has nine genes encoding ACS isoforms that are classified into three types according to the phosphorylation sites in their C-termini. ACS1, ACS2, and ACS6 are the type I ACS isoforms and have phosphorylation sites for mitogen-activated protein kinases (MAPKs) and calcium-dependent protein kinases (CDPKs; Liu and Zhang, 2004; Kamiyoshihara et al., 2010). ACS2 and ACS6 could be regulated by the MAPKs MPK3, and MPK6 at both the transcriptional and posttranslational levels (Liu and Zhang, 2004; Han et al., 2010; Li et al., 2012). Type II ACSs include ACS4, ACS5, ACS8, and ACS9, and have putative CDPK phosphorylation sites, but not MAPK phosphorylation sites, in their C-termini. ACS7 and ACS11 are classified into Type III ACS isoforms, which lack both types of phosphorylation sites. Previous studies have shown that expression of ACS isoforms is tissue-specific, and that different ACS isoforms respond differently to extracellular stimuli (Zarembinski and Theologis, 1994; Wang et al., 2002).

In plants, MAPK cascades, which consist of MAPKKK, MAPKK, and MAPK, play vital roles in development and in a number of stress responses, including those to wounding, pathogen infection, temperature, salinity, drought, osmolarity, ozone, UV irradiation, ROS, and nutrient deficiency (Group et al., 2002; Pedley and Martin, 2005; Zhang et al., 2007; Pitzschke et al., 2009; Rodriguez et al., 2010; Tena et al., 2011). MPK3 and MPK6 can be regulated by different MAPKKs under different stress conditions (Teige et al., 2004; Liu et al., 2007; Takahashi et al., 2007; Wang et al., 2007, 2008, 2010; Xu et al., 2008; Yoo et al., 2008; Zhou et al., 2009). In addition, recent work shows that the MKK9-MPK3/MPK6 cascade is involved in phosphate (Pi) acquisition (Lei et al., 2014). However, the role of MAPKs in regulation of plant responses to Fe deficiency has not been studied.

Using quantitative RT-PCR (qRT-PCR) analysis, we found that the expression of *ACS2, ACS6, ACS7*, and *ACS11* transcripts in both leaves and roots, *ACS8* transcripts in leaves and *ACS9* in roots were up-regulated by Fe deficiency. Further analysis showed that MPK3/MPK6 participates in Fe deficiency-induced ethylene production. Loss function in MPK3 and MPK6 suppressed the expression of ACS2, ACS6, and the Fe deficient responses. As a result, the *mpk3* and *mpk6* plants had a reduced soluble Fe content and severe chlorosis symptoms compared to the wild-type (WT) plants when grown under Fe deficient conditions.

## Materials and methods

### Plant materials

*Arabidopsis thaliana* Columbia (Col-0) ecotype was used as the WT control. T-DNA insertion mutant alleles of *MPK3* (At3g45640), *MPK6* (At2g43790), *ACS2* (At1g01480), *ACS6* (At4g11280) were described previously (Liu and Zhang, 2004; Wang et al., 2007; Han et al., 2010). The high-order *acs* mutants generated in Dr. Athanasios Theologis' laboratory (Tsuchisaka et al., 2009) were obtained from the Arabidopsis Biological Resource Center (ABRC). The stock numbers of the high-order ACS mutants *acs2/acs4/acs5//acs6/acs7/acs9* and *acs1/acs2/acs4/acs5/acs6/acs7/ acs9/acs11* are CS16649 and CS16651, respectively.

### Growth conditions and treatments

For hydroponic experiments, seeds were vernalized at 4°C for 3 days in the distilled water. Then they were sown in 1.5 ml bottom-cut centrifuge tubes containing 400 μL of 0.6% agarose gel. The tubes were held in the holes of a thin polyurethane raft floating on nutrient solution (Lucena et al., 2006). This arrangement allowed the plants growing in the float to uptake the nutrient solution via the agarose gel. The nutrient solution (without Fe) had the following composition: 2000 μM Ca(NO_3_)_2_, 500 μM KH_2_PO_4_, 750 μM K_2_SO_4_, 650 μM MgSO4, 50 μM KCl, 1 μM MnSO_4_, 0.5 μM ZnSO_4_, 0.5 μM CuSO_4_, 10 μM H_3_BO_3_, and 0.05 μM (NH_4_)_6_Mo_7_O_24_. Fe-EDTA was added or not added to the nutrient solution depending on the experiments. The pH of the nutrient solution was adjusted to 6.0. The growth chamber of seedlings was set 22°C day/20°C night temperatures, relative humidity 60%, and a 10 h photoperiod at a photosynthetic irradiance of 300 μmolm^−2^s^−1^ (Lucena et al., 2006).

Experiments using 10 day old seedlings were performed at swimming medium culture as described (Li et al., 2012). After being vernalized at 4°C for 3 days, the surface sterilized seeds were sown in liquid half-strength (1/2) Murashige and Skoog (MS) medium and grown in a growth chamber at 22°C with continuous light(70 μE/m^−2^sec^−1^). Five-day-old seedlings were transferred to 20-ml gas chromatography (GC) vials with 6 ml of liquid 1/2 MS medium (10 seedlings per vial) and the growth conditions maintained the same as before. Seedlings were grown in 1/2 MS swimming medium for 10 days, and then transferred to 1/2 MS medium with or without Fe. Ethylene were measured at 4 day or the indicated day (in the time course analysis) after the treatment, while analysis of transcript abundance was performed at 7 days after treatment.

### RNA isolation and qRT-PCR

According to the manufacturer's instructions (Invitrogen, Carlsbad, CA, USA), Triozol reagent were used for total RNA isolation. To remove the residual genomic DNA, five micrograms of RNA were then treated with RNase-free DNase I (Takara Bio, Tokyo, Japan). Using M-MLV reverse transcriptase, First-strand cDNA was synthesized (Promega, Madison, WI, USA). qRT-PCR was performed as described previously (Zheng et al., 2009). A LightCycler 480 machine was used for PCR amplification of cDNA (Roche Diagnostics, Basel, Switzerland) with SYBR Premix Ex Taq Kit (Takara Bio). Quantitative assays were performed in triplicate on each sample and the reference gene β*-tubulin* was used as an internal control. Transcript levels relative to β*-tubulin* were calculated using the formula 2^−ΔΔCt^. All primer sequences used for the PCR reactions are provided in Supplementary Table 1.

### Protein extraction and immunoprecipitation kinase assay

Protein extraction was performed as described previously (Ren et al., 2002). Total protein was extracted from whole seedlings by grinding in extraction buffer containing 100 mM HEPES, pH 7.5, 5 mM EDTA, 5 mM EGTA, 10 mM Na_3_VO_4_, 10 mM NaF, 50 mM β-glycerophosphate, 10 mM dithiothreitol, 1 mM phenylmethylsulfonyl fluoride, 5 g ml^−1^ leupeptin, 5 g ml^−1^ aprotinin, and 5% glycerol. Supernatants were transferred into 1.5 mL tubes after centrifugation at 18,000 × g for 40 min. Samples should be quickly frozen in liquid nitrogen and stored at −80°C until further analyses. The concentration of protein extracts was determined using the Bio-Rad protein assay kit (Bio-Rad) with bovine serum albumin as a standard (Ren et al., 2002). Twelve micrograms of protein was loaded into each lane and separated by SDS-PAGE.

Immunoprecipitation kinase assay was performed as described (Lee and Ellis, 2007; Tsuda et al., 2009). Anti-p44/42 MAPK (Erk1/2) (Thr202/Tyr204) antibody, which specifically recognizes the dually phosphorylated-pTXpY- motif in phospho-MPK3 and phospho-MPK6, was used to detecting the amount of phosphorylated MPK3 and MPK6, i.e., the activities of the MPK3 and MPK6. The secondary antibody was a horseradish peroxidase-conjugated goat anti-rabbit IgG antibody. The protein membranes were visualized with an Enhanced Chemiluminescence Kit (Roche) and then it was exposed to X-ray film.

### Ethylene measurement

After treatment, the GC vials which contain Arabidopsis seedlings were flushed and capped immediately. Twenty-four hours before the measurement of the ethylene production, the GC vials were flushed with fresh air to remove the ethylene accumulated before the day. Ethylene accumulated in the headspace of the GC vials over a 24 h period were determined by gas chromatography and mass spectrometry at indicated times (Kim et al., 2003; Liu and Zhang, 2004). Then the seedlings were harvested and weighed. Samples were frozen in liquid nitrogen for future analysis.

### Chlorophyll content analysis

The SPAD value (a measure of total chlorophyll content) of the fully expanded youngest leaves was measured by a portable chlorophyll meter (SPAD-502; Konica Minolta Sensing, JP).

### Measurement of soluble Fe concentration

To determine the concentration of soluble Fe in plants, approximately 0.5–1 g of new leaves of treated seedlings were ground in liquid nitrogen. Five volumes of deionized water were added to extract the soluble Fe at room temperature. After centrifugation, the supernatant was collected in new tubes (Zheng et al., 2009). Inductively coupled plasma mass spectrometry (ICP-MS, Agilent 7500ce, Santa Clara, CA, USA) was used for Fe concentration measurement.

## Result

### Effect of Fe deficiency on ethylene production

**Figure 1:**
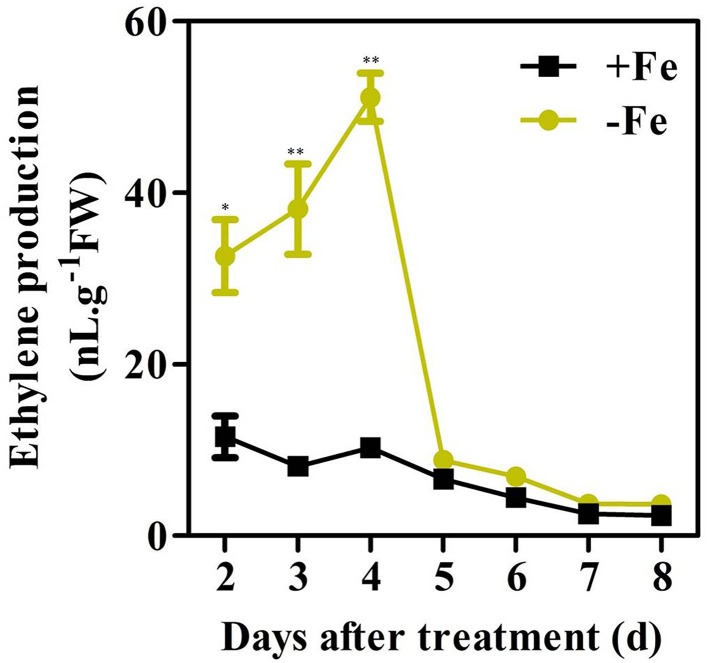
**Time-course analysis of ethylene production in WT plants under Fe-sufficient (+Fe) and -deficient (−Fe) conditions**. Ten-day-old seedlings were transferred to Fe-sufficient (50 μM EDTA-Fe) or Fe-deficient (0 μM EDTA-Fe) nutrient solutions. For each time point shown, ethylene accumulated over a 24 h period from 10 seedlings in the headspace of GC vials was measured. Data are shown as the mean ±SEM (*n* = 3). Columns marked with “^*^” indicate a significant difference (*P* < 0.05), and “^**^” indicate a highly significantly difference (*P* < 0.01).

A previous study found that ethylene production in the roots of the strategy I plants pea, tomato, and cucumber increased under conditions of Fe deficiency (Romera et al., 1999). A time-course experiment was carried out with WT seedlings to investigate whether Fe deficiency induced ethylene production in Arabidopsis (Figure 1). To accurately measure the ethylene production at different treatment time, the GC vials were flushed with fresh air at 24 h prior to the measurement. Ethylene accumulated over a 24 h period was determined at the day indicated. Upon initiation of Fe deprivation, the induction of ethylene production began at the 2nd day and reached its maximum level at the 4th day. The maximum level of ethylene production in Fe deficient conditions was approximately three times higher than that in Fe sufficient conditions. After reaching its maximum level, ethylene levels decreased gradually and dropped to basal levels at 5 days after initiation of Fe deficient conditions.

### Effect of Fe deficiency on ACS transcription

**Figure 2:**
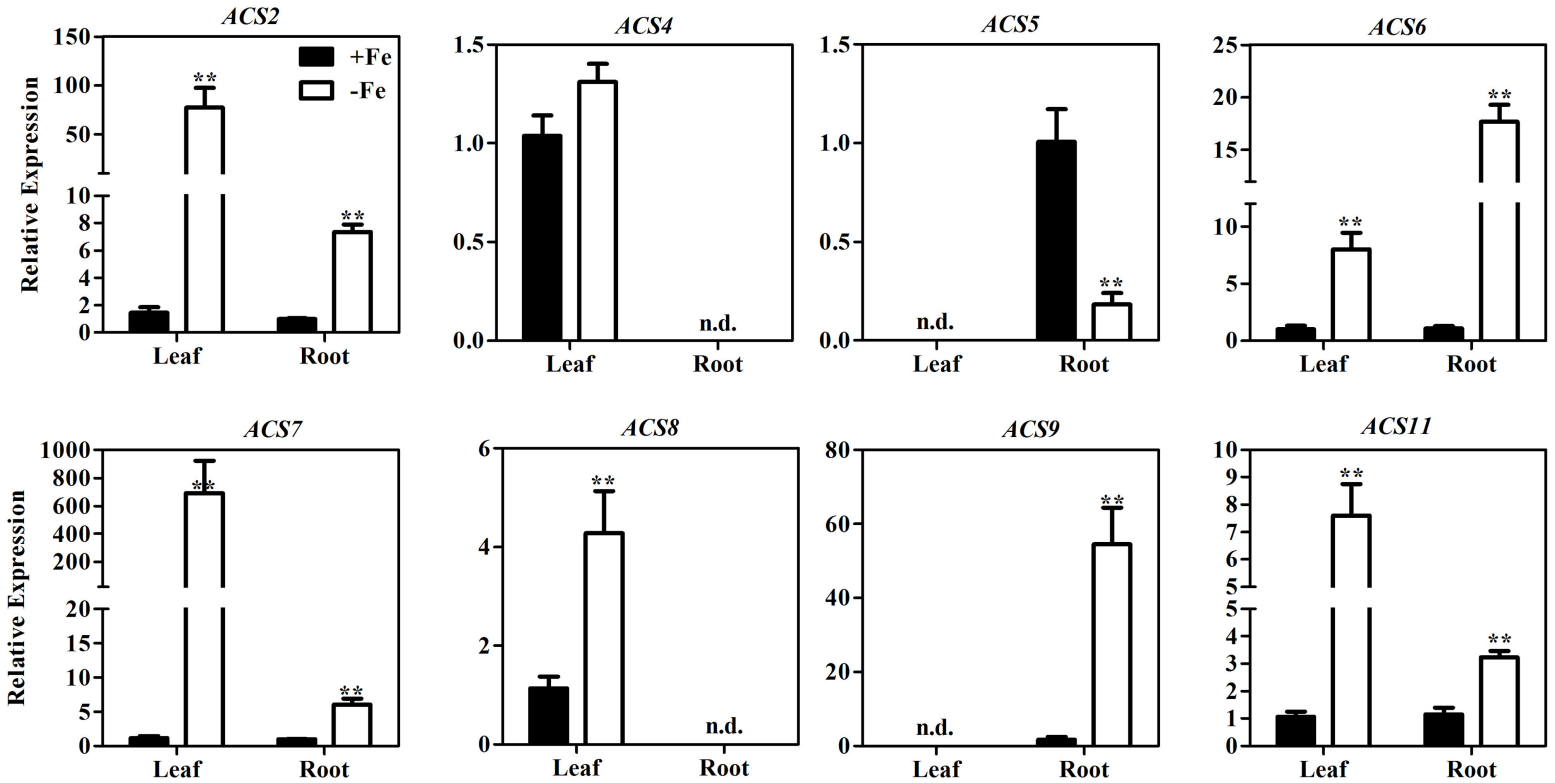
**Transcription levels of *ACS* genes in the leaves and roots of WT seedlings under Fe-sufficient (+Fe) and -deficient (−Fe) conditions**. One-month-old seedlings were transferred to +Fe (50 μM EDTA-Fe) or −Fe (0 μM EDTA-Fe) nutrient solutions for 7 days. Leaves and roots were collected for RNA extraction. All values are expressed relative to the expression level under Fe-sufficient conditions (control—set to 1.0) as appropriate. Data are shown as the mean ±SEM (*n* = 3). Column marked with “**” indicate a highly significantly difference (*P* < 0.01). n.d. indicates “not detectable.”

Using semi-quantitative PCR, a previous study demonstrated that several *ACS* genes, including *ACS4, ACS6*, and *ACS9*, are up-regulated under the stressful conditions of Fe deficiency (García et al., 2010). To identify all the ACS isoforms involved in ethylene production under Fe deficiency, we used qRT-PCR to measure the expression of all nine *ACS* genes in Arabidopsis grown under Fe-sufficient and Fe-deficient conditions. We were able to detect the expression of eight of the nine *ACS* genes (all but *ACS1*) in leaf, root, or both (Figure 2). The transcript abundance of *ACS2, ACS6, ACS7*, and *ACS11* increased significantly in both leaf and root after 7 days of Fe deprivation. *ACS8* transcripts were detected only in leaf, and were four-fold more abundant under Fe deficient conditions than under Fe sufficient conditions. While *ACS4* transcripts were also detected only in leaf, Fe deficiency did not lead to a significant change in *ACS4* mRNA. *ACS5* mRNA and *ACS9* mRNA were detected only in root. *ACS9* expression was over 30-fold higher in conditions of Fe deficiency compared to those of Fe sufficiency, whereas expression of *ACS5* was reduced. These results suggest that *ACS2, ACS6, ACS7, ACS8, ACS9*, and *ACS11* may contribute to ethylene induction under conditions of Fe deficiency.

### Mutation of *ACS2* suppressed ethylene production induced by Fe deficiency

**Figure 3:**
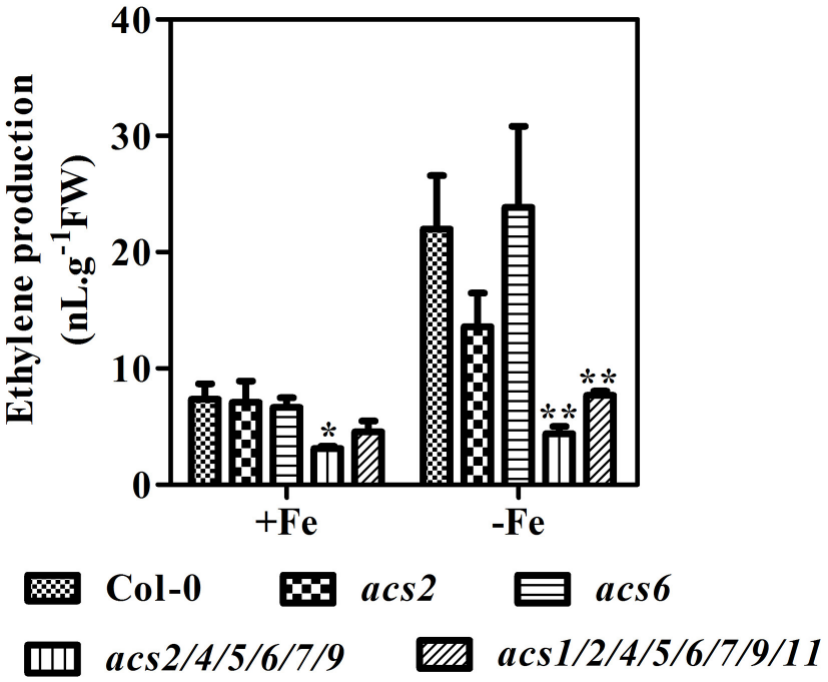
**Ethylene production of WT, *acs* mutant plants under Fe-sufficient (+Fe) and -deficient (−Fe) conditions**. Ten-day-old seedlings were transferred to Fe-sufficient (50 μM EDTA-Fe) or -deficient (0 μM EDTA-Fe) nutrient solutions. Ethylene accumulated over a 24 h period from 10 seedlings in the headspace of GC vials was measured at 4 day after the treatment. Data are shown as the mean ±SEM (*n* = 3). Columns marked with “^*^” indicate a significant difference (*P* < 0.05), and “^**^” indicate a highly significantly difference (*P* < 0.01).

As the rate-limiting enzyme in ethylene biosynthesis, ACS is well positioned to influence ethylene production. To explore the involvement of ACS isoforms in Fe deficiency-induced ethylene production, *acs2, acs6*, and high-order *acs* mutants were used. In these mutants, expression of corresponding *ACS* genes was abolished (Supplementary Figure 1A). Under conditions of Fe deficiency, *acs2* seedlings produced ethylene at a level that was only 60% of that in WT seedlings (Figure 3). Surprisingly, *ACS6* gene mutation did not affect Fe-induced ethylene induction, as *acs6* seedlings produced the same amount of ethylene as the WT. Ethylene production in high-order *acs* mutants (*acs2/acs4/acs5/acs6/acs7/acs9* and *acs1/acs2/acs4/acs5/ acs6/acs7/acs9/acs11*) was very low in both Fe sufficient and Fe deficient conditions.

### Mutation of *ACS2* suppressed the upregulation of Fe deficiency-responsive genes

**Figure 4:**
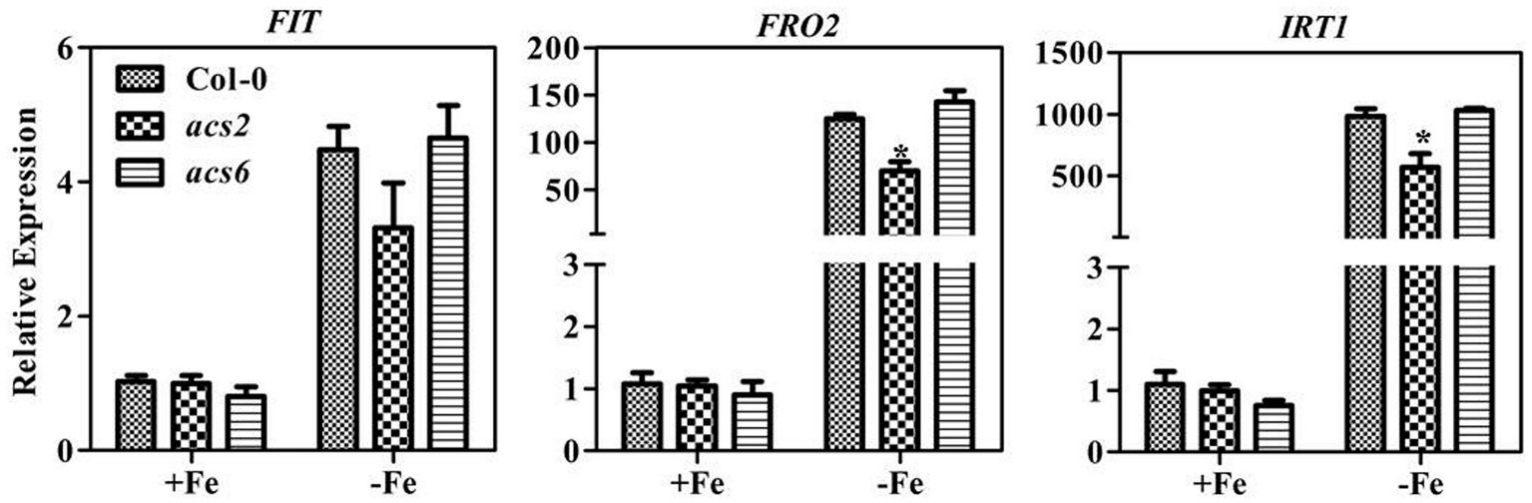
**Transcription levels of Fe-deficiency responsive genes in *acs2* and *acs6* mutant plants**. Ten day old seedlings cultured in swimming medium were transferred to Fe-deficient medium for 7 days and sampled for RNA extraction. All values are expressed relative to the expression level under Fe-sufficient conditions (control—set to 1.0) as appropriate. Data are shown as the mean ±SEM (*n* = 3). Column marked with “*” indicate a significant difference (*P* < 0.05).

Ethylene regulates expression of *FER* (or *FER*-like) and thereby regulates gene expression of downstream Fe transporter, ferric reductase, and H^+^-ATPase (Lucena et al., 2006). We did not observe obvious phenotypic differences between WT and *acs2* or *acs6* mutants in Fe sufficient or deficient conditions (Supplementary Figure 2A). However, in conditions of Fe deficiency, upregulation of Fe deficiency-responsive genes was attenuated in *acs2* mutants (Figure 4) and the high order ACS mutant (Supplementary Figure 3 Specifically, expression levels of *FIT, FRO2*, and *IRT1* genes in *acs2* mutants were reduced 40, 50, and 33% from levels in WT seedlings, respectively. In contrast, the induction of Fe deficiency-responsive gene expression did not change in the *acs6* mutants. To confirm the reduction of FRO2 expression indeed affected the Ferric-chelate reductase (FCR) activity, FCR assay was performed on *acs2, acs6*, and high order ACS mutants. Compared to the WT plants, the FCR activity in *acs2* was slightly reduced, but not significant (Supplementary Figure 2B). In contrast, FCR activity in the high order ACS mutants were significantly reduced. The FCR activity is in agreement with the *FRO2* transcript level.

### Fe deficiency activates MPK3/MPK6 at both transcript and protein levels

**Figure 5:**
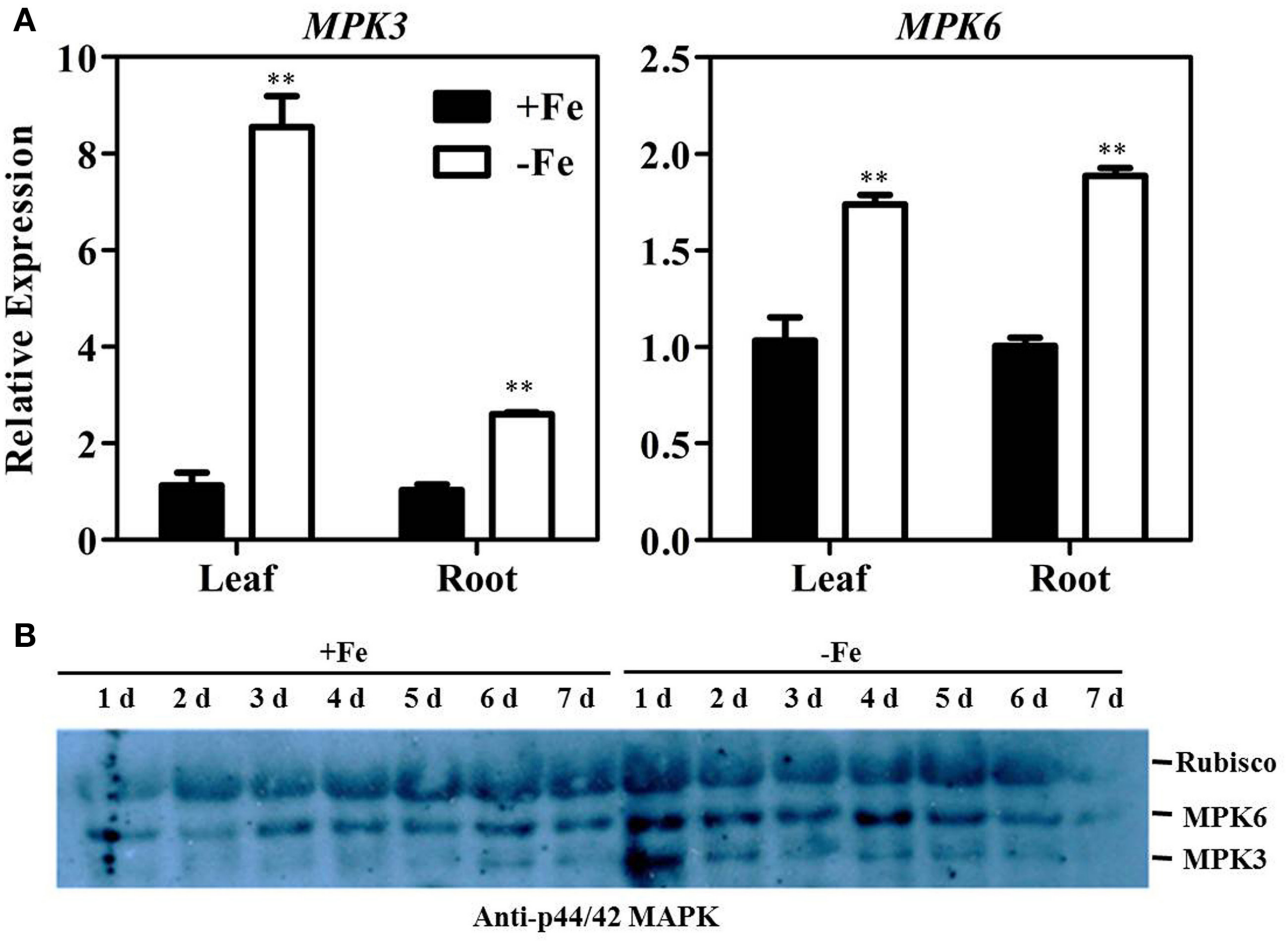
**Fe deficiency activates MPK3 and MPK6. (A)** Transcription levels of MPK3 and MPK6 in the leaf and root of WT seedlings under Fe-sufficient (50 μM EDTA-Fe) and -deficient (0 μM EDTA-Fe) conditions. All values are expressed relative to the expression level under Fe-sufficient conditions (control—set to 1.0) as appropriate. Data are shown as the mean ±SEM (*n* = 3). **(B)** Immunoprecipitation kinase assay of MPK3 and MPK6 activity using Anti-p44/42 MAPK antibody. Ten-day-old seedlings were transferred to Fe-sufficient or -deficient nutrient solutions. Column marked with “**” indicate a highly significantly difference (*P* < 0.01).

Previous research has shown that MPK3/MPK6-mediated phosphorylation of ACS2 and ACS6 proteins leads to their stabilization and accumulation (Liu and Zhang, 2004; Han et al., 2010; Li et al., 2012). qRT-PCR results confirmed that *ACS2* and *ACS6* transcript levels are increased under Fe deficiency conditions (Figure 2). To further understand the roles of MPK3 and MPK6 in Fe deficiency-induced ethylene production, both transcript and enzymatic activity levels of MPK3 and MPK6 were determined. Results showed that levels of *MPK3* and *MPK6* transcripts were significantly increased under Fe deficiency (Figure 5A). Specifically, expression of *MPK3* was induced eight-fold in leaf and 2.5-fold in root, whereas that of *MPK*6 was induced only 1.8-fold in leaf and 2.8-fold in root. Furthermore, immunoprecipitation kinase assay was performed using anti-p44/42 MAPK (Erk1/2) (Thr202/Tyr204) antibody to detect the amount of phosphorylated MPK3 and MPK6, (Figure 5B). While the activation of MPK6 persisted 4–5 days after treatment, the activation of MPK3 persisted only 1–2 days after treatment. Taken together, these results indicate that the transcript abundance and enzymatic activities of MPK3 and MPK6 were induced by Fe deficiency.

### Loss of *MPK3* or *MPK6* resulted in reduction of ethylene production and the expression level of *ACS2* and *ACS6* under Fe deficiency

**Figure 6:**
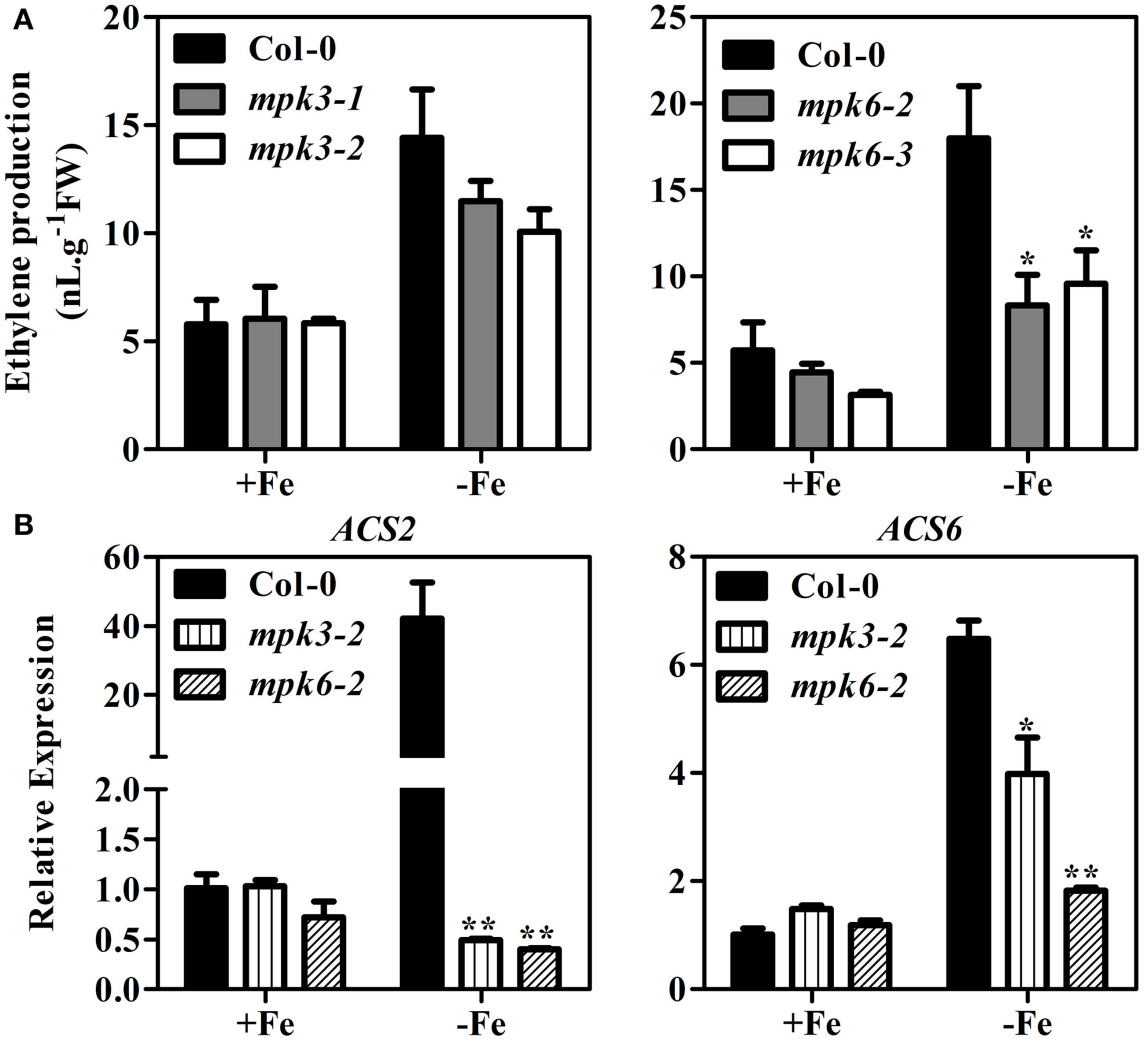
**Ethylene production and expression of *ACS2* and *ACS6* in *mpk3* and *mpk6* mutants. (A)** Ethylene production in *mpk3* and *mpk6* mutants. **(B)** Transcript abundances of *ACS2* and *ACS6* genes in *mpk3* and *mpk6* mutants. Ten-day-old seedlings were transferred to Fe-sufficient (50 μM EDTA-Fe) or -deficient (0 μM EDTA-Fe) nutrient solutions. Ethylene accumulated over a 24 h period from 10 seedlings in the headspace of GC vials was measured at 4 days after the treatment. qRT-PCR was conducted using RNA extracted from whole seedlings at 7 days after –Fe treatment. Data are shown as the mean ±SEM (*n* = 3). Columns marked with “^*^” indicate a significant difference (*P* < 0.05), and “^**^” indicate a highly significantly difference (*P* < 0.01).

To further demonstrate that the MPK3/MPK6 cascade participates in Fe-induced ethylene production, two *mpk* mutants for each MPK were used in subsequent experiments: *mpk3-1, mpk3-2, mpk6-2*, and *mpk6-3*. Phosphorylation enzymatic activity assay showed that the mutants lost the function of the corresponding kinase activity (Supplementary Figure 1B). Fe deficiency-induced ethylene production in *mpk3-1* and *mpk3-2* was reduced by 15 and 28%, respectively, compared to that of WT seedlings (Figure 6A). In *mpk6* mutants, reduction of Fe deficiency-induced ethylene biosynthesis was much more severe. Compared to WT seedlings, both *mpk6* mutants maintained only 50% of ethylene production. These results provide further support for the involvement of MPK3 and MPK6 in ethylene production under Fe deficiency.

ACS2 and ACS6 are known to be regulated by the MPK3/MPK6 at both transcriptional level and posttranslational level (Li et al., 2012). To determine whether mutation in MPK3 and MPK6 would affect the expression ACS2 and ACS6, qRT-PCR was performed in the *mpk3* and *mpk6* plants grown under Fe sufficient or deficient conditions. While Fe deficiency induced the expression of *ACS2* and *ACS6*, the up-regulation was suppressed in the *mpk3* and *mpk6* mutants (Figure 6B).

### The *MPK3* and *MPK6* mutants showed severe chlorosis under Fe deficiency

**Figure 7:**
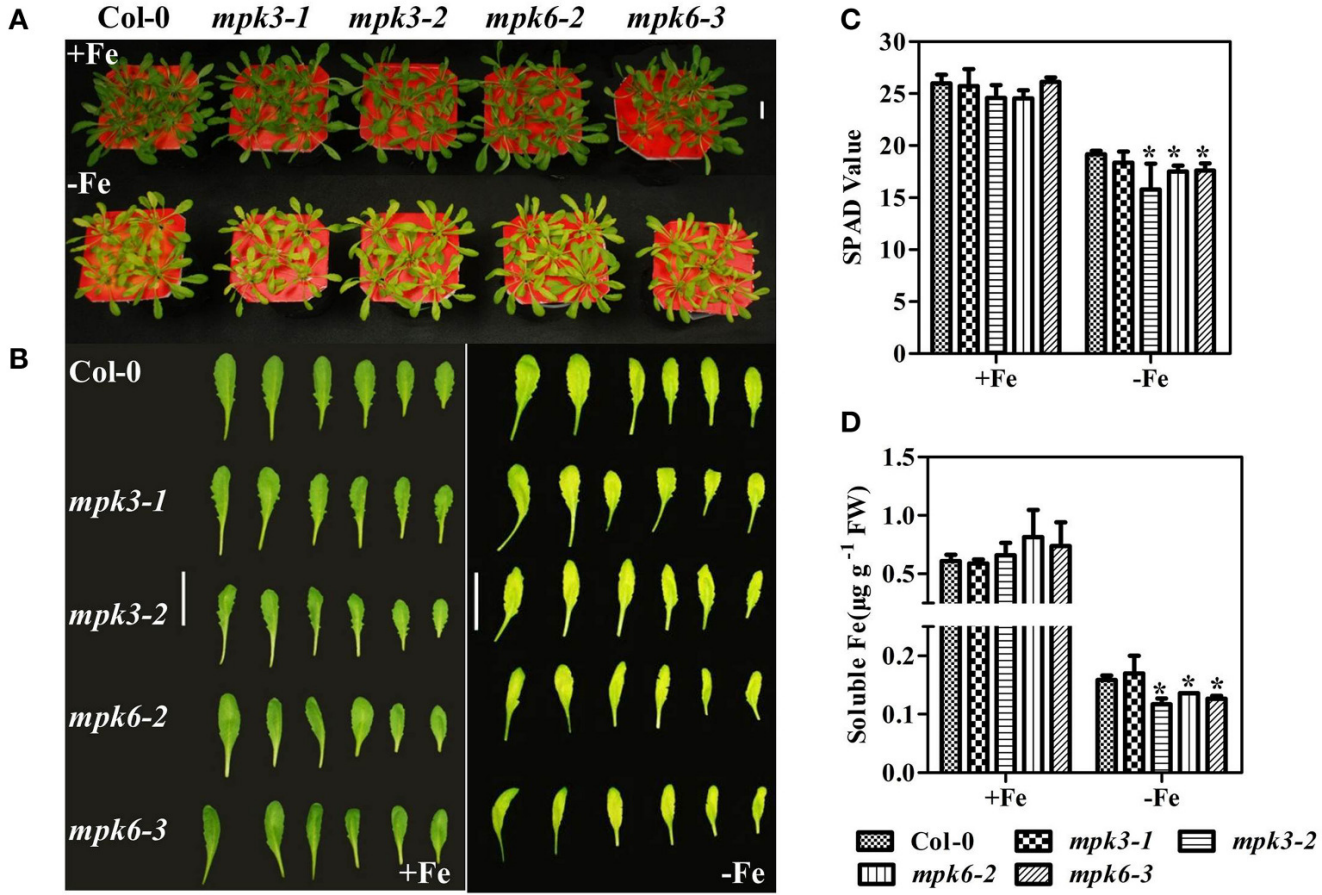
**Phenotypes of *mpk3* and *mpk6* mutants grown in Fe-sufficient or Fe-deficient conditions. (A)** Growth performance of WT, *mpk3*, and *mpk6* mutant plants. 30 day-old seedlings were grown in Fe -sufficient (50 μM) or -deficient (0 μM) nutrient solution for 7 days. **(B)** Young leaves (from the third to the eighth) were detached to display their phenotypes. **(C)** SPAD values of WT, *mpk3*, and *mpk6* mutants. **(D)** Soluble Fe concentration of WT, *mpk3*, and *mpk6* mutants. Scale bar = 3 cm. Data are shown as the mean ±SEM (*n* = 3). Column marked with “*” indicate a significant difference (*P* < 0.05).

To further investigate the role of MPKs in Fe deficiency, the growth performance and the expression of the Fe deficiency-responsive genes were investigated in the *mpk* mutants. Under Fe sufficient conditions, growth performance of WT seedlings was not significantly different from that of mutants (Figures 7A,B). However, under Fe deficient conditions, the mutants, especially the *mpk3-1* and *mpk6-3* mutants, were smaller and showed more severe chlorosis than WT seedlings (Figures 7A,B). Consistent with the mutants' chlorosis phenotypes, chlorophyll content, measured as the leaf SPAD value, was lower in *mpk3-1, mpk6-2*, and *mpk6-3* mutants than in WT seedlings (Figure 7C).

**Figure 8:**
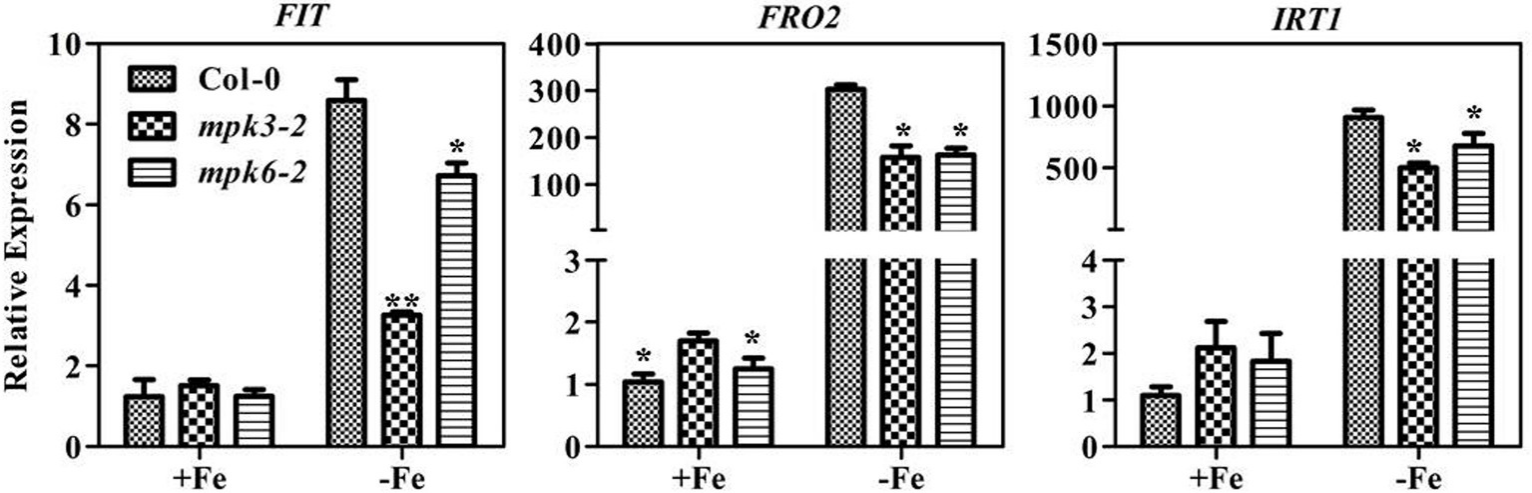
**Transcription levels of Fe-deficiency responsive genes in *mpk3* and *mpk6* mutants**. Ten day old seedlings cultured in swimming medium were transferred to Fe-deficient medium for 7 days and sampled for RNA extraction. All values are expressed relative to the expression level under Fe-sufficient conditions (control—set to 1.0) as appropriate. Data are shown as the mean ±SEM (*n* = 3). Columns marked with “^*^” indicate a significant difference (*P* < 0.05), and “^**^” indicate a highly significantly difference (*P* < 0.01).

To determine whether the severe chlorosis phenotype in the *mpk3* and *mpk6* mutants was due to a decrease in leaf Fe content, the total leaf Fe concentrations in the mutants were measured. Fe concentrations were at the same level in WT seedlings and mutants (Supplementary Figure 4). To further investigate the relationship between Fe content and SPAD value, soluble Fe concentrations in the leaves of mutants and WT seedlings were determined. Our results showed that soluble Fe in *mpk3-2* and both *mpk6* mutants was marginally lower than that in the WT (Figure 7D). Furthermore, Fe deficiency-responsive gene expression was suppressed in both the *mpk3-2* and *mpk6-2* mutants compared to WT seedlings (Figure 8). Specifically, the induction of *FRO2* and *IRT1* in both mutants dropped to nearly 50% of WT levels. In the *mpk3-2* mutant, expression of *FIT* dropped to 30% of that in WT seedlings.

## Discussion

In strategy I plants, ethylene production is induced by Fe deficiency stress (Romera et al., 1999; Romera and Alcantara, 2004), which in return, functions as a positive regulator of Fe deficiency response (Romera and Alcantara, 1994; Li and Li, 2004; Lucena et al., 2006; Waters et al., 2007; García et al., 2010; Lingam et al., 2011; Romera et al., 2011). The aim of the study was to explore the regulatory mechanism of this physiological response. In the report, we demonstrated the involvement of MPK3 and MPK6 in the Fe-deficient induced ethylene production based on the following evidence. Firstly, the transcript abundance and enzymatic activities of MPK3 and MPK6 increased under Fe deprivation condition (Figure 5). ACS2 and ACS6 are known to be regulated by the MPK3/MPK6 at both transcriptional level mediated by a transcription factor and posttranslational level via the direct protein phosphorylation by MPK3/MPK6 (Li et al., 2012). The up-regulated expression of *ACS2, ACS6* in Fe-deficient plants at both root and leaf tissues (Figure 6B) is likely a consequence of up-regulation of MPK3 and MPK6. Secondly, the Fe-deficient induced ethylene production decreased in the *mpk3* and *mpk6* mutants (Figure 6A). Thirdly, the expression of Fe acquisition genes, *FIT, FRO2*, and *IRT1* in *mpk3* and *mpk6* mutants was less up-regulated by Fe deprivation than that in the WT (Figure 8). As a result, the *mpk3* and *mpk6* plants had a reduced soluble Fe content and severe chlorosis symptoms compared to the WT plants when grown under Fe deficient conditions (Figure 7).

The Fe-deficient induced ethylene production was not completely abolished in *mpk3, mpk6, acs2*, and *acs6* mutants (Figure 3, Figure 6). It suggests that other ACS isoforms are also involved in the process. Indeed, other than *ACS2* and *ACS6*, the expression of *ACS7, ACS9*, and *ACS11* genes in roots, and *ACS7, ACS8*, and *ACS11* in leaves were also up-regulated by Fe deficiency (Figure 1). Changes in the expression of the above ACS isoforms should also contribute to the Fe-deficient induced ethylene production. García et al. (2010) examined the expression of *ACS4, ACS6, ACS9*, and *ACS11* in Arabidopsis roots in response to Fe deficiency. They found that the expression of *ACS4, ACS6*, and *AC9* were up-regulated by Fe-deficiency. In contrast to that, the expression of *ACS4*, which was upregulated by Fe-deficiency in that research (García et al., 2010), was not detected in the study. In addition, the increased expression of *ACS11* in this study was not detected by García et al. (2010). The inconsistency between the two studies may be attributed to the different experimental conditions used. While García et al. (2010) examined the expression of *ACS* genes at 24 h after –Fe treatment, we did qRT-PCR on the plant tissues that had been treated with -Fe deficiency for 7 days. Seven days of Fe deprivation was chosen as that is when chlorotic symptoms are visible (Supplementary Figure 5). According to Vert et al. (2003), the level of *IRT1* and *FRO2* transcripts increases at 3 d and reaches to the maximum level at 5 d after –Fe treatment. In their paper, the *IRT1* and *FRO2* transcript abundance at day 7 is similar to day 3 of Fe deficiency (Vert et al., 2003). To verify that, we did a time-course qRT-PCR analysis using plant samples with different period of –Fe treatment, including 1, 3, 5, and 7 days of Fe-deprived treatment. Results showed that the up-regulation levels of the tested -Fe induced genes were stable from day 1 to day 7 of the treatment (Supplementary Figure 6). Thus, the expression of Fe acquisition related genes in response to 7 days of Fe deficiency is a good reflection of the transcript abundance for the genes measured. As to the expression of the *ACS* genes, after 7 days of –Fe treatment, the up-regulation of rapid response *ACS* genes may have returned to a normal level. On the other hand, the long term Fe deficiency may turn on the general stress responsive genes, which is not directly related to Fe deficiency. Whether ACS4 and ACS11 are response to short or long term Fe-deficiency differently needs to be further investigated.

In summary, we demonstrated that the MPK3/MPK6 participates in Fe deficiency-induced ethylene production. Loss function in MPK3 and MPK6, or their downstream ACS2 isoform suppressed the Fe deficient responses. Roles of the other Fe-responsive ACS isoforms, such as ACS7, ACS 9, and ACS11 at leaves and roots, and ACS8 in leaves, in Fe deficiency-induced ethylene production remained to be explored. Additional studies, including studies of the regulation of upstream genes of MPK3/MPK6 in Fe deficiency-induced ethylene production and of other ACS isoforms would expand our understanding of the regulatory mechanisms of ethylene induction under Fe deficiency.

### Conflict of interest statement

The authors declare that the research was conducted in the absence of any commercial or financial relationships that could be construed as a potential conflict of interest.
