# Supplementary material for: MPK3/MPK6 are involved in iron deficiency-induced ethylene production in Arabidopsis
Source: Front Plant Sci. 2015 Nov 3;6:953. doi: 10.3389/fpls.2015.00953 (PMC4630569; doi:10.3389/fpls.2015.00953)
Supplement: Supplementary file 1 [file DataSheet1.DOCX]

Supplementary Material

# MPK3/MPK6 are involved in iron deficient induced ethylene production in Arabidopsis

**Lingxiao Ye^1^, Lin Li^1^, Lu Wang^1,2^, Shoudong Wang^1^, Juan Du^1^, Shuqun Zhang^1^, Huixia Shou^1^***

**^1^**State Key Laboratory of Plant Physiology and Biochemistry, College of Life Sciences, Zhejiang University, Hangzhou, China

^2^ Tea Research Institute, Chinese Academy of Agricultural Sciences, Hangzhou, China

***Correspondence：**Huixia Shou, State Key Laboratory of Plant Physiology and Biochemistry, College of Life Sciences, Zhejiang University, 866 Yuhangtang Rd, Hangzhou, 310058, China.

[huixia@zju.edu.cn](mailto:huixia@zju.edu.cn)

# Supplementary Table

**Supplementary Table 1.** Primer sequence of genes in Arabidopsis for qRT-PCR

| Genes | Primers | |
| --- | --- | --- |
| *AtTublin*    *AtFIT*    *AtIRT1*    *AtFRO2*  *AtbHLH38*    *AtbHLH100*  *AtACS2*    *AtACS4*  *AtACS5*    *AtACS6*  *AtACS7*  *AtACS8*    *AtACS9*  *AtACS11*  *AtMPK3*  *AtMPK6* | fwd  rev fwd  rev fwd  rev fwd  rev fwd  rev fwd  rev fwd  rev fwd  rev fwd  rev fwd  rev fwd  rev fwd  rev fwd  rev fwd  rev fwd  rev fwd  rev | CGACAATGAAGCTCTCTACGA AAGTCACACCGCTCATTGTT  GGAGAAGGTGTTGCTCCATC TCCGGAGAAGGAGAGCTTAG  AAGCTTTGATCACGGTTGG TTAGGTCCCATGAACTCCG  CTTGGTCATCTCCGTGAGC AAGATGTTGGAGATGGACGG  AGCAGCAACCAAAGGCG CCACTTGAAGATGCAAAGTGTAG  AAGTCAGAGGAAGGGGTTACA GATGCATAGAGTAAAAGAGTCGCT  AAATCTTAAGGCATAACGTCT TAAAAGTTACCATTTGACCCC  GCTTCCCACGCCATATTATCC CGCCATTTTAGATCCCTATCA  GACTCTCATGTTTTGCCTTGC TTGGAAGCCATTAGAGCTTGA  GTTCCAACCCCTTATTATCC CCGTAATCTTGAACCCATTA  ACATTAGGATAAACCGGGAA CCGTCTTTAGTTTTCTTTTCGAG  CTCAATATCTCTCCCGGTTCT TTCTAGTAGGCGACGGTCCAT  CATACCTCGACGAAAACCAGA TCATGTCAACCCAACAGAACA  CTGAGAGAGAGAAAGGATAGG TTGTCCATAAAGAATGCTCGG  GCCCTTAGCTAAACTTTTCTC CGTGCAATTTAGCAAGGTACT  CCACCTTATCCTCGCCAATCC ATGGGCCAATGCGTCTAAAAC |

**Supplementary Figure 1. Transcript abundance of *ACS2, ACS6* and the activities of MPK3, MPK6 in the corresponding mutants. (A)Transcription level of *ACS2* and *ACS6* in WT and mutants.** 10 days old seedlings were used for qRT-PCR analysis. Data are shown as the mean ±SEM (n=3). Columns marked with one asterisk indicates a significant difference (P < 0.05), and double asterisks indicate a highly significantly difference (P < 0.01). **(B)** **Immunoblot analysis of MPK3 and MPK6 activity in WT and mutants.** 10 days old seedlings were used for western blot analysis. Twelve μg protein in each lane is separated by SDS-PGAE.


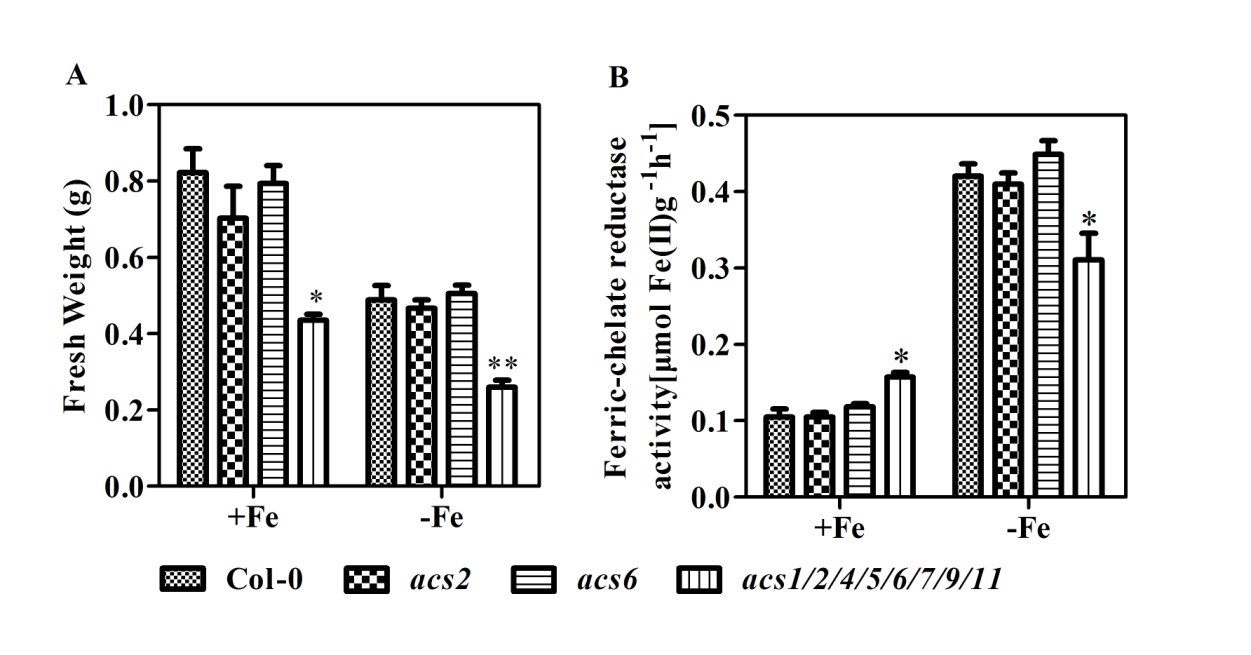


**B**

**Supplementary Figure 2.** **Fresh weight (A) and ferric-chelate reductase activity (B) of WT, *acs2*, *acs6* and *acs1/2/4/5/6/7/9/11 mutant* plants.** Thirty days old seedlings were transferred to nutrient solutions with or without EDTA-Fe for 7 days, and then the fresh weight of the seedlings and Ferric-chelate reductase in roots were measured. Data are shown as the mean ±SEM (n=3). Columns marked with one asterisk indicates a significant difference (P < 0.05), and double asterisks indicate a highly significantly difference (P < 0.01).

**Supplementary Figure 3. Transcription levels of Fe-deficient genes in the roots of WT and *acs1/2/4/5/6/7/9/11* mutant plants.** 10-day-old seedlings cultured in swimming media were transferred to Fe-deficient media for 7 days. Data are shown as the mean ±SEM (n=3). Columns marked with one asterisk indicates a significant difference (P < 0.05), and double asterisks indicate a highly significantly difference (P < 0.01).


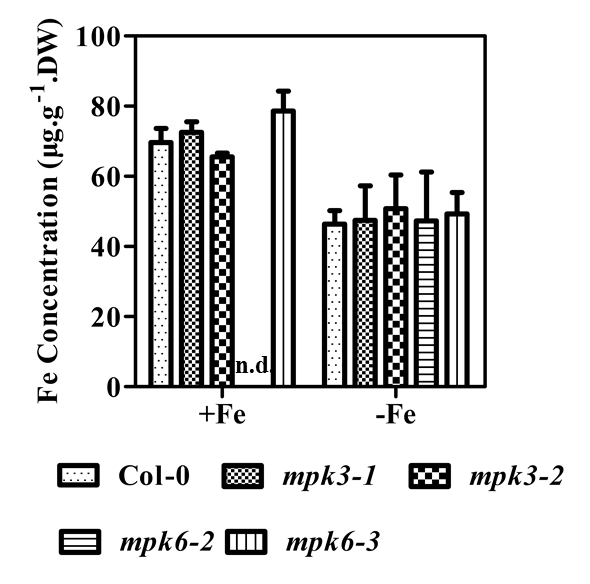


**Supplementary Figure 4.** **Total iron concentration of WT, *mpk3* and *mpk6* mutants.** 30 day-old seedlings were grown in Fe -sufficient (50 μM) or -deficient (0 μM) nutrient solution for 7 days. Data are shown as the mean ±SEM (n=3). n.d. indicates “not detectable”.

**
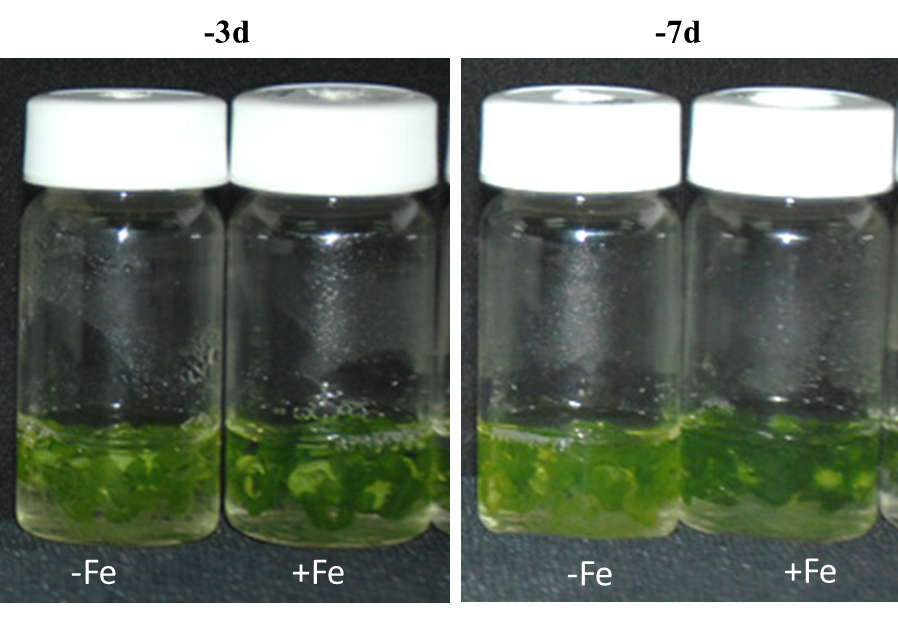
**

**Supplementary Figure 5. Growth performance of seedlings cultured in swimming media.** Ten-day-old seedlings were transferred to Fe-sufficient (50 μM EDTA-Fe) or -deficient (0 μM EDTA-Fe) nutrient solutions.





**Supplementary Figure 6. Time course analysis of the expression of Fe-deficient responsive genes.** Ten-day-old seedlings cultured in swimming medium were transferred to Fe-deficient medium for 7 days. All values are expressed relative to the expression level under Fe-sufficient conditions (control-set to 1.0) as appropriate. Whole seedlings were collected for RNA extraction at 0, 1, 3, 5, 7 days after treatment.
